# Polyhexamethylene biguanide functionalized cationic silver nanoparticles for enhanced antimicrobial activity

**DOI:** 10.1186/1556-276X-7-267

**Published:** 2012-05-24

**Authors:** Sumaira Ashraf, Nasrin Akhtar, Muhammad Afzal Ghauri, Muhammad Ibrahim Rajoka, Zafar M Khalid, Irshad Hussain

**Affiliations:** 1 National Institute for Biotechnology and Genetic Engineering (NIBGE), Jhang Road, Faisalabad, 38000, Pakistan; 2 Department of Bioinformatics and Biotechnology, Government College University, Allama Iqbal Road, Faisalabad, 38000, Pakistan; 3 Department of Chemistry, School of Science & Engineering (SSE), Lahore University of Management Sciences (LUMS), DHA, Lahore Cantt, 54792, Pakistan

**Keywords:** Cationic silver nanoparticles, Polyhexamethylene biguanide, Antimicrobial activity

## Abstract

Polyhexamethylene biguanide (PHMB), a broad spectrum disinfectant against many pathogens, was used as a stabilizing ligand for the synthesis of fairly uniform silver nanoparticles. The particles formed were characterized using UV-visible spectroscopy, FTIR, dynamic light scattering, electrophoretic mobility, and TEM to measure their morphology and surface chemistry. PHMB-functionalized silver nanoparticles were then evaluated for their antimicrobial activity against a gram-negative bacterial strain, *Escherichia coli*. These silver nanoparticles were found to have about 100 times higher bacteriostatic and bactericidal activities, compared to the previous reports, due to the combined antibacterial effect of silver nanoparticles and PHMB. In addition to other applications, PHMB-functionalized silver nanoparticles would be extremely useful in textile industry due to the strong interaction of PHMB with cellulose fabrics.

## Background

Antimicrobial activity of silver nanoparticles (Ag NPs) is well documented, and they are currently being used in a variety of commercial antimicrobial products including textile products and paints etc. [1-6]. Although the mechanism of their antimicrobial activity is not completely understood yet, silver, whether in the form of ionic or metallic state, is known to destabilize and increase the permeability of bacterial membranes [7]. It results in the collapse of the plasma membrane potential, disruption of ion transport processes, and the depletion of the levels of intracellular ATP [8-10]. Silver also inactivates essential respiratory enzymes and proteins responsible for RNA and DNA replication in bacteria [7,11,12]. Ag NPs can penetrate inside the microbial cells, damaging sulfur and phosphorus containing compounds such as DNA and proteins [9,13-15]. Although Ag NPs form complexes with various amino acids to inhibit protein's function, their toxicity to the mammalian cells is very limited, thus, warranting their antimicrobial applications in medical and healthcare products [16-20].

**Figure 1:**
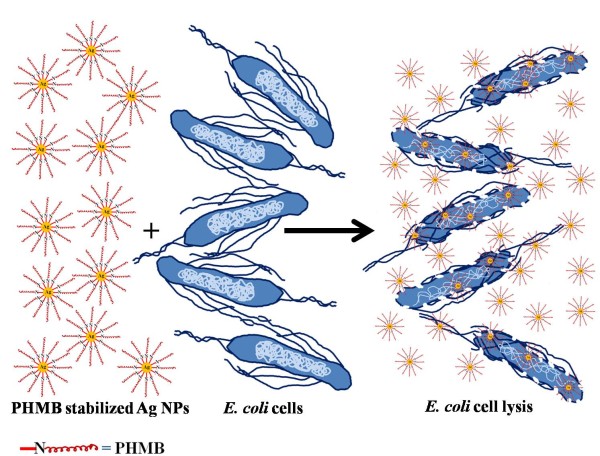
The interaction of PHMB-functionalized silver nanoparticles with the bacteria to demonstrate their antibacterial activity.

Due to the growing interest in the use of Ag NPs to develop antimicrobial products, various methods are being developed for their preparation with a fair control over their size, shape, and the surface chemistry [5,21-26]. Recent studies have shown that antimicrobial properties of Ag NPs are significantly affected by their nanoscale dimensions (size and shape) and the surface ligands/charge, and thus, the efforts are being made to produce Ag NPs with the highest possible antimicrobial properties to minimize the load of nanoparticles in any commercial products without compromising their antimicrobial activity [5,21-26]. One such elegant approach has recently been reported to integrate the antimicrobial properties of an enzyme, lysozyme, with those of silver nanoparticles, and the resulting hybrid nanoparticles showed improved microbial toxicity over other preparations [27]. In order to increase the repertoire of effective antimicrobial products, we have now integrated the antimicrobial property of polyhexamethylene biguanide (PHMB) with that of silver nanoparticles, and to the best of our knowledge, the antimicrobial activity of these hybrid silver nanoparticles is much higher than the previous reports. PHMB is a positively charged polymer having polymeric biguanide units in the backbone of its structure [−(CH_2_)_6_.NH.C(=NH).NH.C(=NH).NH-]_*n*_, where *n* ranges from 2 to 40 having an average value of 11 [28]. It is being used in medicine [29-35], textile, and food industry [36,37] as an antiseptic agent since many years due to its broad spectrum antibacterial effect. PHMB acts as a bacteriostatic if administered at low concentrations, i.e., from 1 to 10 μg/mL and as bactericidal at high doses, i.e., ≥10 μg/mL [38-40]. It damages the membrane integrity by nonspecifically interacting with acidic lipid residues of cytoplasmic membrane [41,42]. PHMB also affects nucleic acids due to its strong interaction with them causing structural and functional changes in the microbial genome [43]. Keeping in view the widespread antimicrobial applications of PHMB, we set out to coat it on silver nanoparticles to develop a hybrid product with enhanced antimicrobial properties. Using *Escherichia coli* as a model microorganism, the bacteriostatic and bactericidal concentrations of these cationic hybrid silver nanoparticles were found to be 0.075 and 0.15 μg/mL, respectively, which are much higher than the previous reports on silver nanoparticles. Figure 1 shows a simple cartoon to demonstrate the antibacterial activity of PHMB-functionalized silver nanoparticles.

## Methods

PHMB (20% *w*/*v*) was purchased from Arch Chemicals (Norwalk, CT, USA). All other chemicals were purchased from Sigma-Aldrich (St. Louis, MO, USA), except tryptone, agar, and yeast extract which were purchased from MP Biomedicals (Santa Ana, CA, USA), and were used as received without further purification. Pure strain of *E. coli* was provided by Invitrogen (Carlsbad, CA, USA). Luria Bertani (LB) medium, used for the growth study of *E. coli*, was prepared using 1% tryptone, 0.5% yeast extract, and 1% sodium chloride in water, and pH was adjusted to 7 using 0.5 M solution of sodium hydroxide and hydrochloric acid. Ultrapure water with a resistivity of 18.2 MΩ cm was used as the solvent in all preparations.

### Synthesis of Ag NPs

In a typical experiment to prepare PHMB functionalized Ag NPs, a given volume of PHMB aqueous solution, starting from 0.5 to 200 μg/mL (ideally, 15 mL, 20 μg/mL), was added to an aqueous solution of AgNO_3_ (25 mL, 1 mM) under vigorous stirring. The reaction mixture was stirred vigorously for 2 h to let PHMB form a complex with silver. Freshly prepared aqueous solution of sodium borohydride (5 mL, 0.8 mg/mL) was then added quickly to PHMB-Ag solution, and the reaction was allowed to complete for 5 h. The yellow/brownish suspension of silver nanoparticles was then filter purified to remove excess PHMB and other impurities using centrifuge filters (Amicon® Ultra centrifugal filters by Millipore Corporation, Billerica, MA, USA ) having a molecular weight cutoff value of 30 KDa and then stored at room temperature for further analysis and use in subsequent experiments.

### Antimicrobial activity of PHMB-functionalized Ag NPs

In order to study the effect of PHMB-stabilized Ag NPs on bacterial growth, *E. coli* strain was grown on an agar plate, and its fresh colony was transferred with the help of a sterilized loop into a shake flask which was then incubated in an incubator shaker for 12 h at 37°C at a shaking speed of 150 rpm under aerobic conditions. The growth of bacteria was measured by taking optical density (OD) of samples at 600 nm. When the OD of growth culture approached 1 at 600 nm, the bacterial culture was then transferred into new shake flasks at a density of 2.5% bacteria into each flask. Different concentrations of clean PHMB-coated Ag NPs were then added in triplicate starting from 0.075 to 0.15 μg/mL of Ag NPs (different tested concentrations of Ag NPs were 0.075, 0.09, 0.1, 0.12, 0.135, and 0.15 μg/mL) into shake flasks just before inoculating with *E. coli*. Same concentrations of Ag NPs were also added into the LB media for the purpose of control experiment to serve as a blank while measuring their OD at 600 nm. All these shake flasks containing *E. coli* strains and Ag NPs, along with controls, were placed in an incubator shaker for 24 h at 37°C at a shaking speed of 150 rpm. The growth of bacteria was monitored by measuring OD of samples at regular intervals at 600 nm. The bacterial growth curves were then plotted against time based on the data obtained by carefully monitoring the bacterial growth. Based on these growth curves, the effect of silver nanoparticles on specific growth rate and doubling time of *E. coli* was observed.

### Characterization

Transmission electron microscopy of Ag NPs was carried out using a high-resolution transmission electron microscope (JEOL, JEM-3010, JEOL Ltd., Akishima, Tokyo, Japan) operating at 300 kV. Nanoparticle specimens, for inspection by transmission electron microscopy, were prepared by slow evaporation of one drop of a dilute aqueous solution of the particles on a carbon-coated copper mesh grid. ImageJ software was used to calculate the particle size distribution from transmission electron micrographs. The particle size distribution and surface potential of nanoparticles were determined using Zetasizer Nano ZS (Malvern Instruments, Westborough, MA, USA). The surface chemistry of Ag NPs was determined using Bruker Alpha-P FTIR (Bruker Optik Gmbh, Ettlingen, Germany) with a diamond ATR attachment. UV-visible spectrum of Ag NPs suspension was recorded using an Agilent 8453 UV-visible spectrophotometer (Agilent Technologies Inc., Santa Clara, CA, USA). The approximate concentration of Ag NPs was determined using fast sequential atomic absorption spectrometer (AA240FS) by Varian Inc., Palo Alto, CA, USA.

## Results and discussion

Antimicrobial activity of Ag NPs is well known and is believed to be dependent on the size [21,22], shape [5,23,24], surface bound ligands [25,26], and their subsequent surface charge [44]. Recently, it was reported that the surface charge of Ag NPs is the most important factor in this regard while studying the effect of neutral, positively, and negatively charged Ag NPs on the growth of gram-positive bacteria belonging to *bacillus* species [44]. Negatively charged Ag NPs were found to be the most effective to retard the growth of these bacteria. In this study, we have prepared Ag NPs which are positively charged and, in addition, are functionalized with a known antimicrobial agent, PHMB, to enhance the antimicrobial properties of Ag NPs towards gram-negative bacteria.

**Figure 2:**
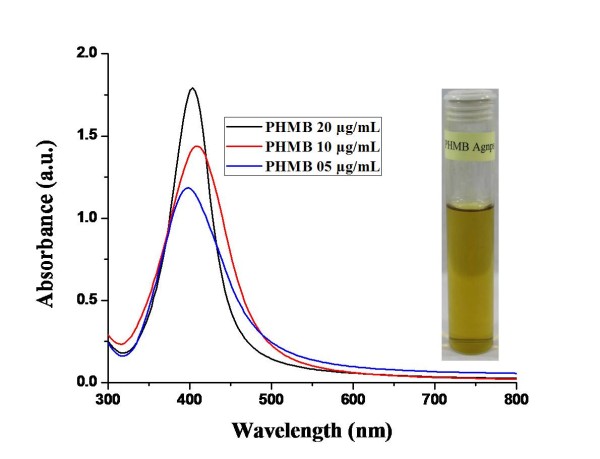
**UV-visible absorption spectra of silver nanoparticles produced using different concentrations of PHMB.** The inset shows the optical image of dispersed silver nanoparticles.

**Figure 3:**
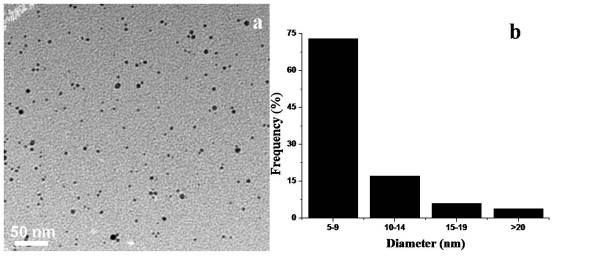
Transmission electron micrograph of PHMB-stabilized silver nanoparticles (a) and the histogram of their size distribution (b).

Since smaller Ag NPs are considered to have higher antimicrobial activity [21,22], we produced Ag NPs using borohydride-reduction method in the presence of PHMB. The so-formed particles were fairly uniform as indicated by their narrow surface plasmon resonance (SPR) bands shown in Figure 2. The size of Ag NPs, however, varied a bit by varying the concentration of PHMB, and the most uniform nanoparticles were obtained using 15 mL of 20 μg/mL PHMB, while keeping other reaction conditions constant, as indicated by their narrowest SPR band (Figure 2). Using lesser concentration of PHMB, the yield of nanoparticles decreased and their polydispersity increased as indicated by a decrease in the intensity and broadening of their SPR bands. By increasing the concentration of PHMB, the PHMB-Ag complex precipitated forming a milky suspension before the addition of reducing agent. Transmission electron micrographs of Ag NPs under optimum conditions, shown in Figure 3a, show that they are fairly round and uniform in size. In order to calculate the size and size dispersity of Ag NPs, we measured the size of at least 300 particles using an ImageJ program, and their histogram shows that about 75% of these particles are in the size range *ca.* 5 to 9 nm (Figure 3b). Dynamic light scattering (DLS) characterization showed that the majority of the particles were of *ca.* 60 to 140 nm in size (see in Additional file 1: Figure S1). The larger size is because DLS measures the hydrodynamic diameter of the particles which includes the size of the capping ligands and the solvent shell.

**Figure 4:**
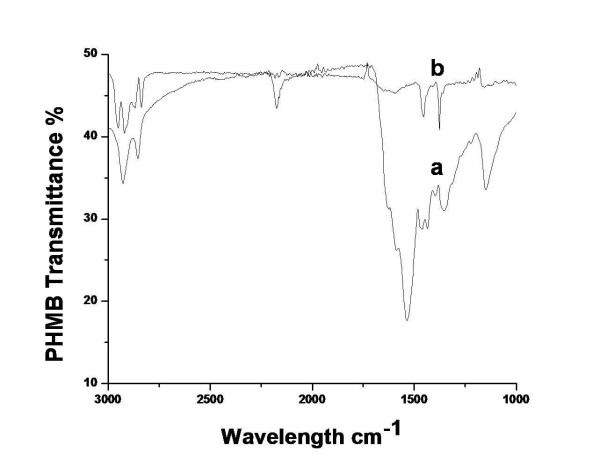
FTIR spectra of PHMB (a) and PHMB-stabilized silver nanoparticles (b).

After the formation of Ag NPs, they were purified using ultracentrifugation filters to ensure the complete removal of excess PHMB. The complete removal of excess PHMB was confirmed by analyzing the filtrate using FTIR spectroscopy, and centrifuge filtration was repeated unless no characteristic IR transmission peaks of PHMB were observed. In order to confirm that PHMB remains bound to the clean nanoparticles' surface, we measured the surface potential of clean particles which was found to be +53 mV indicating the presence of positively charged PHMB on nanoparticles' surface (see in Additional file 1: Figure S2). In order to further confirm the functionalization of silver nanoparticles with PHMB, they were characterized using FTIR spectroscopy which is a valuable tool to characterize various functional groups in organic molecules. PHMB shows sharp spectral peaks between 1,200 and 1,700 cm^−1^ as shown in its FTIR spectrum (Figure 4). Its strong absorption peak centered at 1,536 cm^−1^ is disappeared in PHMB-modified Ag NPs indicating the interaction of imine groups of PHMB with the nanoparticles.

After confirming the presence of PHMB on the surface of clean Ag NPs, we set out to determine their antimicrobial activity. Ag NPs are well known for their antimicrobial activity, and their bacteriostatic and bactericidal concentrations are usually found in the range of 5 and 50 μg/mL, respectively [5,7,25,45-50]. In order to improve the antimicrobial activity and broaden its spectrum, we conjugated Ag NPs with an antimicrobial agent, PHMB. PHMB, if used alone, shows bactericidal activity beyond 10 μg/mL [38]. The bacteriostatic and bactericidal activities of PHMB-functionalized Ag NPs were, however, improved to be 0.075 and 0.150 μg/mL, respectively.

**Figure 5:**
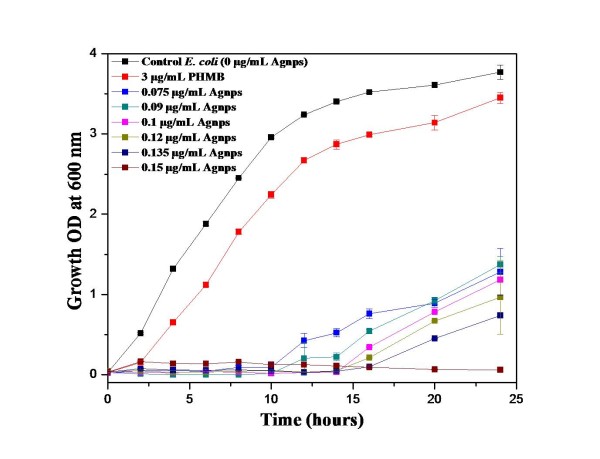
**Effect of the concentration of PHMB-stabilized silver nanoparticles on the growth of *E. coli*.**

Figure 5 shows the growth curves of *E. coli* in the absence and presence of varying concentrations (0.075 to 0.15 μg/mL) of PHMB-functionalized Ag NPs. It is evident from these growth curves that *E. coli* was unable to grow for about 10 h even in the presence of the lowest concentration (0.075 μg/mL) of PHMB-functionalized Ag NPs used. Even after 10 h, the growth rate was very slow, and the total bacterial biomass produced after 24 h was less than one third of that produced in control experiment containing no Ag NPs. The bacterial biomass was estimated by taking optical density of the samples withdrawn at regular intervals during bacterial growth. The growth curves also indicate that *E. coli* was unable to grow in the presence of 0.15 μg/mL or higher concentration of PHMB-functionalized Ag NPs even after 24 h. At this bactericidal concentration of Ag NPs, the black aggregates of *E. coli* were clearly visible floating in the growth media showing the aggregation of bacteria. The aggregation of bacteria at bactericidal concentration of many antimicrobial agents is already known and is usually attributed to the charge neutralization of bacterial cell's surface in case of PHMB [24]. These results thus show more than 100 times increase in the bacteriostatic and bactericidal activities of these cationic Ag NPs compared to the previous reports on Ag NPs. It is worth noticing that PHMB, when used alone (3 μg/mL) as a control, had no significant effect on the trend of bacterial growth which is in accordance with the previous finding [38].

Based on the bacterial growth in the presence and absence of PHMB-functionalized Ag NPs, the specific growth rate (increase in cell mass per unit time, *μ* (per hour)) and doubling time *t*_d_ (hours) for *E. coli* were also calculated (Table 1). It is evident from the values of specific growth rate and doubling time for *E. coli* that maximum specific growth rate (0.92 ± 0.03 h^−1^) and minimum doubling time (0.75 ± 0.02 h) were observed in the absence of functionalized Ag NPs. However, the specific growth rate was decreased and doubling time increased by increasing the concentration of PHMB-functionalized Ag NPs. In the presence of PHMB alone (3 μg/mL), the effect on the specific growth rate and doubling time of *E. coli* was not much significant as shown in Table 1.

**Table 1:** Effect of concentrations of silver nanoparticles

**Concentration of Ag NPs in *E. coli *culture (μg/mL)**	**Specific growth rateμ (h^**-1**^)**	**Doubling timet_**d **_(h)**
0	0.92 ± 0.03	0.75 ± 0.02
0.075	0.25 ± 0.01	2.8 ± 0.01
0.090	0.20 ± 0.01	3.4 ± 0.01
0.100	0.197 ± 0.01	3.52 ± 0.01
0.120	0.18 ± 0.01	4.31 ± 0.01
0.135	0.15 ± 0.01	4.6 ± 0.01
0.150	0.11 ± 0.01	6.3 ± 0.02
PHMB alone (3 μg/mL)	0.788 ± 0.03	0.879 ± 0.02

## Conclusions

To summarize, a cationic biocide (PHMB) was used for the synthesis of fairly uniform silver nanoparticles. The cationic silver particles thus formed showed much higher antibacterial activity against *E. coli* than the previous reports. PHMB is routinely used in textile industry due to its antimicrobial activity and affinity to the cellulose fabrics. Therefore, these PHMB-coated silver nanoparticles with enhanced antimicrobial activity may have very useful applications in textile industry.

## Competing interests

The authors declare that they have no competing interests.

## Authors’ contributions

SA and IH conceived and designed all the experiments. SA performed all the experiments. NA and MAG helped SA while performing antimicrobial experiments of silver nanoparticles against *Escherichia coli*. MIR helped SA while performing statistical analysis and interpretation of antimicrobial effect of Ag NPs. ZMK co-supervised SA, under the supervision of IH, during all studies. SA and IH discussed the interpretation of results. SA and IH co-wrote the draft paper and were involved in all revision process. Correspondence and requests for materials should be addressed to IH. All authors read and approved the final manuscript.

## Supplementary Material

Additional file 1 **Figures S1. and S2.** Description: Figure S1, histogram of the distribution of the hydrodynamic diameter of PHMB-stabilized silver nanoparticles determined by dynamic light scattering. Figure S2, zeta potential of PHMB-stabilized silver nanoparticles based on electrophoretic mobility of PHMB-stabilized silver nanoparticles. Click here for file
