# Supplementary material for: Polyhexamethylene biguanide functionalized cationic silver nanoparticles for enhanced antimicrobial activity
Source: Nanoscale Res Lett. 2012 May 24;7(1):267. doi: 10.1186/1556-276X-7-267 (PMC3492125; doi:10.1186/1556-276X-7-267)
Supplement: Additional file 1 — Figures S1. and S2. Description: Figure S1, histogram of the distribution of the hydrodynamic diameter of PHMB-stabilized silver nanoparticles determined by dynamic light scattering. Figure S2, zeta potential of PHMB-stabilized silver nanoparticles based on electrophoretic mobility of PHMB-stabilized silver nanoparticles. [file 1556-276X-7-267-S1.doc]

Polyhexamethylene Biguanide Functionalized Cationic Silver Nanoparticles for Enhanced Antimicrobial Activity

*Sumaira Ashraf,^a, c^ Nasrin Akhtar,^a^ Muhammad Afzal Ghauri,^a^ Muhammad Ibrahim Rajoka,^a, b^ Zafar M. Khalid,^a^ and Irshad Hussain^c*^*

^a^ National Institute for Biotechnology and Genetic Engineering (NIBGE), Jhang road, Faisalabad, Pakistan. ^b^ Department of Bioinformatics and Biotechnology, Government College University, Allama Iqbal Road, Faisalabad, Pakistan. ^c^ Department of Chemistry, School of Science & Engineering (SSE), Lahore University of Management Sciences (LUMS), DHA, Lahore Cantt – 54792, Pakistan.

* Corresponding author: Professor Irshad Hussain

Email: [ihussain@lums.e du.pk](mailto:ihussain@lums.e du.pk); [irshadnibge@gmail.com](mailto:irshadnibge@gmail.com)

Tel: +92 42 3560 8133

Fax: +92 42 3560 8314

**Abstract**

Polyhexamethylene biguanide (PHMB), a broad spectrum disinfectant against many pathogens, was used as a stabilizing ligand for the synthesis of fairly uniform silver nanoparticles. The particles formed were characterized using UV-visible spectroscopy, FTIR, dynamic light scattering (DLS), electrophoretic mobility and TEM to measure their morphology and surface chemistry. PHMB-functionalized silver nanoparticles were then evaluated for their antimicrobial activity against a gram negative bacterial strain, *E. coli*. These silver nanoparticles were found to have about 100 times higher bacteriostatic and bactericidal activities, compared to the previous reports, due to the combined antibacterial effect of silver nanoparticles and PHMB. In addition to other applications, PHMB-functionalized silver nanoparticles would be extremely useful in textile industry due to the strong interaction of PHMB with cellulose fabrics.

**Keywords**: Cationic silver nanoparticles, polyhexamethylene biguanide (PHMB), antimicrobial activity.


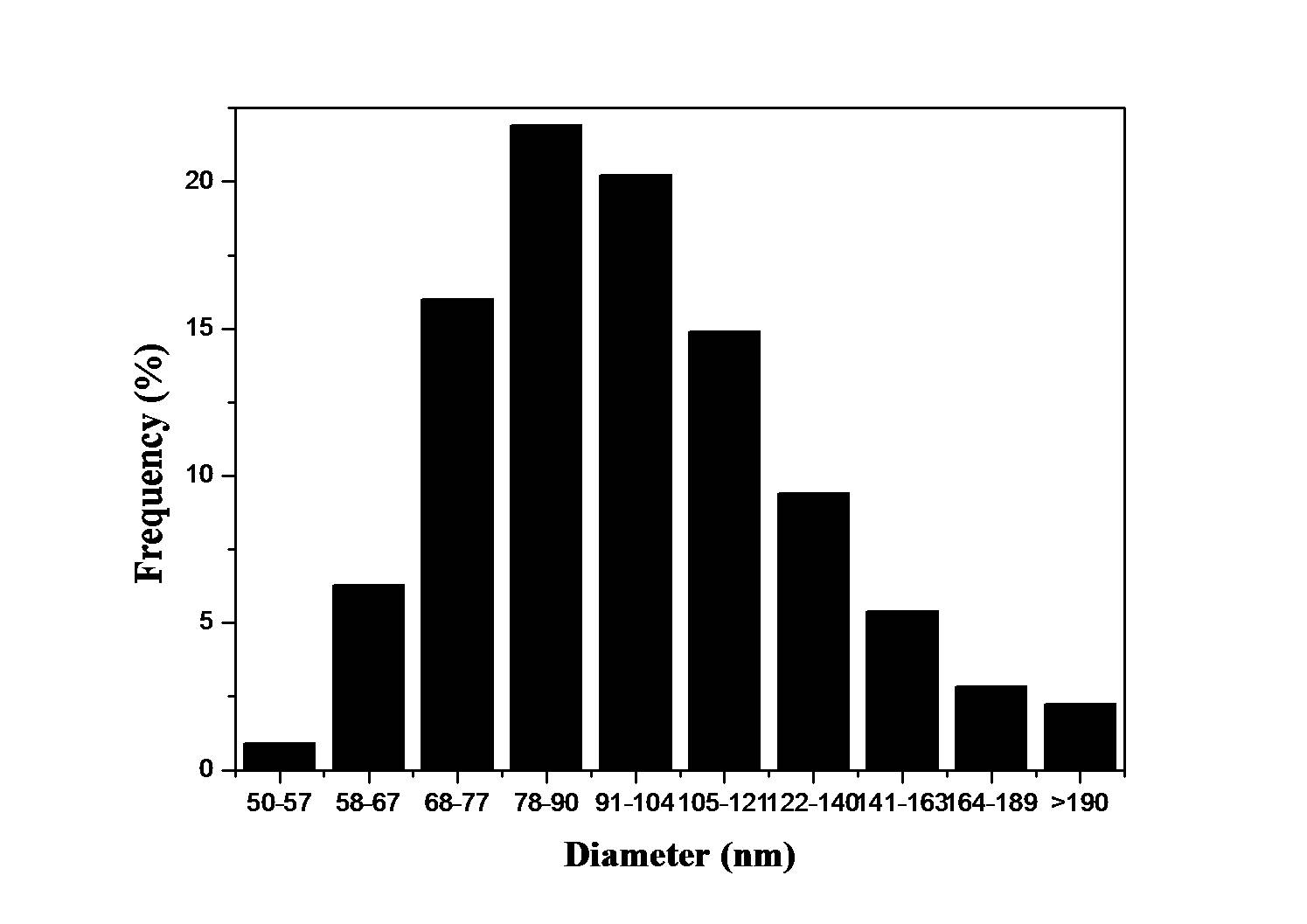


**Figure S1**. Histogram of the distribution of the hydrodynamic diameter of PHMB - stabilized silver nanoparticles determined by dynamic light scattering.


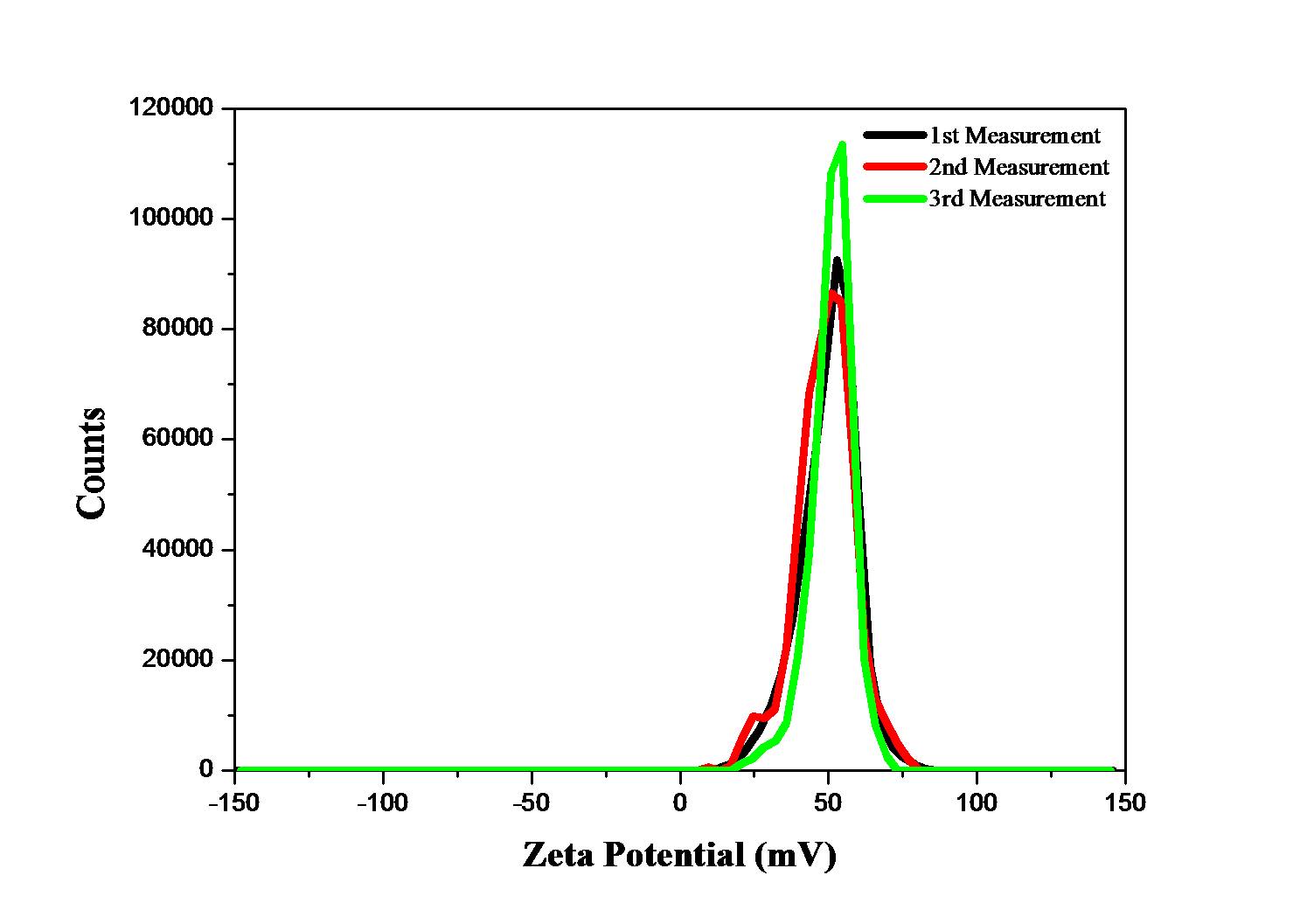


**Figure S2**. Zeta potential of PHMB - stabilized silver nanoparticles based on electrophoretic mobility of PHMB-stabilized silver nanoparticles.
